# Effect of sofosbuvir-based DAAs on changes in lower-density lipoprotein in HCV patients: a systematic review and meta-analysis

**DOI:** 10.1186/s12879-021-06657-9

**Published:** 2021-09-21

**Authors:** Ying-Wen Wang, Wei-Ping Lee, Yi-Hsiang Huang, Ming-Chih Hou, Keng-Hsin Lan

**Affiliations:** 1 Healthcare and Management Center, Taipei Veterans General Hospital, Taipei, Taiwan, ROC; 2 Department of Medical Research, Taipei Veterans General Hospital, Taipei, Taiwan, ROC; 3 Institute of Biochemistry and Molecular Biology, College of Life Sciences, National Yang Ming Chiao Tung University, Taipei, Taiwan, ROC; 4 Division of Gastroenterology and Hepatology, Department of Medicine, Taipei Veterans General Hospital, 201, Section 2, Shi-Pai Road, Taipei, 112 Taiwan, ROC; 5 School of Medicine, College of Medicine, National Yang Ming Chiao Tung University, Taipei, Taiwan, ROC; 6 Institute of Clinical Medicine, College of Medicine, National Yang Ming Chiao Tung University, Taipei, Taiwan, ROC; 7 Institute of Pharmacology, College of Medicine, National Yang Ming Chiao Tung University, Taipei, Taiwan, ROC

**Keywords:** Direct-acting antivirals, Hepatitis C virus, Lipids, Low-density lipoprotein, Sofosbuvir

## Abstract

**Background:**

Previous studies reported worsened lipid profiles in patients infected with hepatitis C virus (HCV) during direct-acting antivirals (DAAs) treatment. This study aimed to investigate the effect of sofosbuvir (SOF)-based DAAs on changes in low-density lipoprotein (LDL) in HCV patients.

**Methods:**

A systematic review of articles published before 31 May 2021 was conducted by searching MEDLINE, Cochrane Library, EMBASE, and CINAHL Plus. Eligible studies were those comparing SOF-based DAAs and non-SOF DAAs for HCV patients and providing numerical data for changes in LDL. Risk of Bias in Non-randomized Studies- of Interventions was used for assessing risk of bias, and meta-analysis was performed for changes in LDL.

**Results:**

Six studies comprising 1248 patients were included, 848 patients treated with SOF-based DAAs and 400 patients with non-SOF DAAs vs. SOF-based DAAs group had significantly greater increases in LDL from baseline to week 4 than non-SOF DAAs group (P = 0.001). However, changes in LDL from baseline to the end of treatment (P = 0.060), to post-treatment week 12 (P = 0.263), and to post-treatment week 24 (P = 0.319) did not significantly differ between the two groups. Further comparison of SOF/ledipasvir with asunaprevir/daclatasvir revealed a similar trend in changes in LDL.

**Conclusions:**

For HCV patients, SOF-based DAA regimens were associated with rapid and significant increases in LDL during the initial 4 weeks of treatment, and the changes did not sustain after the end of treatment. Potential mechanism might be related to the phosphoramidate side chain of SOF.

**Supplementary Information:**

The online version contains supplementary material available at 10.1186/s12879-021-06657-9.

## Background

Hepatitis C virus (HCV) is one of the major causes of liver-related morbidity and mortality [1], estimatedly infecting approximate 180 million people worldwide [2]. HCV life cycle is initiated by virus particles binding to hepatocellular receptors, endocytosis, acidification of endosome, HCV envelope glycoproteins fusing with endosomal membranes, and viral genome releasing into cytoplasm [3]. Subsequently, internal ribosome entry site-mediated translation of viral ribonucleic acid (RNA) enables viral gene expression and processing [4]. HCV RNA is translated to generate a large polyprotein, which is processed by both host proteases and viral proteases (HCV NS2/3 and NS3/4A) to produce structural proteins for viral assembly and nonstructural protein for RNA replication. HCV NS4B, NS5A, and NS5B constitute a complex for RNA replication, which occurs in the membranous webs [5]. Following replication, genomic RNA in complex with NS5A transit to lipid droplets, where core protein localizes and virion assembly occurs [6]. After acquiring apolipoprotein B, apolipoprotein E, very low-density lipoprotein (VLDL), and low-density lipoprotein (LDL), HCV infectious particles egress in a manner that parallels the VLDL secretion pathway [7, 8].

Patients infected with HCV have a twofold higher prevalence of hepatic steatosis than hepatitis B virus patients, suggesting a clear correlation between HCV infection and non-alcoholic fatty liver disease [9, 10]. HCV patients are also more likely to have decreased serum levels of apolipoprotein B-bearing lipoproteins because HCV captures these lipoproteins [11].

Currently, direct-acting antivirals (DAAs) have replaced interferon as the standard treatment for HCV infection [12, 13]. According to mechanisms of action and therapeutic targets, DAAs are classified into four categories: NS3/4A protease inhibitor, NS5A replication complex inhibitor, and NS5B nucleoside and non-nucleoside polymerase inhibitor [14]. In the class of NS5B nucleoside polymerase inhibitor, sofosbuvir (SOF) is the only drug and plays an important role in the combination of other DAAs for HCV treatment [12, 13]. HCV virion is tightly associated with hepatocyte-derived lipoproteins to form a lipid-laden particle, called lipo-viro-particle. It was thought that HCV captures lipoproteins that are released to blood after viral clearance by DAAs. Given that the concept is true, all DAA regimens would have similar effects on lipid profiles. However, several studies reported worsened lipid profiles in HCV patients during DAAs treatment and SOF-based DAAs seemed to have greater effect on LDL elevation [15, 16]. In the study by Meissner et al. the patients treated with SOF/ribavirin had significantly increased levels of low-density lipoprotein (LDL) from baseline to the end of treatment and to post-treatment week 48 [15]. Younossi et al. also found significantly increased LDL from baseline to the end of treatment and to post-treatment week 4 in the HCV patients treated with SOF/ledipasvir (LDV) [16]. Because the mechanism underlying the interaction between SOF-based DAAs and changes in LDL is still unclear, we performed a systematic review and meta-analysis to investigate the effect of SOF-based DAAs on changes in LDL in HCV patients.

## Methods

### Search strategy

This systematic review and meta-analysis was conducted in accordance with the Preferred Reporting Items for Systematic Reviews and Meta-Analyses guidelines [17]. MEDLINE, Cochrane Library, EMBASE, and CINAHL Plus were searched until 31 May 2021. The following search terms were used: (direct acting antiviral OR asunaprevir OR boceprevir OR daclatasvir OR dasabuvir OR elbasvir OR glecaprevir OR grazoprevir OR ledipasvir OR ombitasvir OR paritaprevir OR pibrentasvir OR simeprevir OR sofosbuvir OR telaprevir OR velpatasvir OR voxilaprevir) AND (hepatitis C OR HCV) AND (lipid OR cholesterol OR HDL OR LDL OR triglyceride OR lipoprotein). The reference lists of the relevant studies were also searched manually to identify additional studies.

### Selection criteria

The inclusion criteria of the systematic review were: (a) comparative study; (b) patients were infected with HCV; (c) one of the patient groups was treated with DAAs and one of the DAAs was SOF; (d) one of the patient groups was treated with non-SOF DAAs; (e) numerical data were provided for LDL for quantitative analysis. Non-original articles (e.g. letters, comments, editorials, reviews, or case reports) were excluded.

### Study selection and data extraction

Based on the search strategy, two independent reviewers performed literature searches to identify eligible studies and a third reviewer was consulted for any uncertainty regarding eligibility. The following information was extracted from studies that met the eligibility criteria: the name of the first author, year of publication, study design, demographic data, genotype, DAAs regimens, duration of treatment, percentage of patients achieving sustained virologic response (SVR), fibrosis 4 index, baseline level of HCV RNA, exclusion criteria, and levels of LDL at baseline, week 4, post-treatment week 12, and post-treatment week 24.

### Risk of bias

We used the Risk Of Bias In Non-randomized Studies-of Interventions (ROBINS-I) to assess the included studies [18]. Quality assessment was also performed by the independent reviewers and a third reviewer was consulted for any uncertainties. The ROBINS-I tool assesses bias across seven domains including: bias due to confounding, bias in selection of participants into the study, bias in classification of interventions, bias due to deviations from intended interventions, bias due to missing data, bias in measurement of outcomes, and bias in selection of the reported result. Risk of bias for each domain is categorized as low, moderate, serious, critical risk of bias, and no information. An overall judgment on risk of bias across the seven domains is then determined.

### Statistical analysis

For the primary endpoint, changes in LDL, means and standard deviations were calculated and were compared between the patients treated with SOF-based DAAs and those with non-SOF DAAs. Because SOF/LDV and asunaprevir (ASV)/daclatasvir (DCV) were the most prevalent regimens in the included studies, meta-analysis was also performed for comparing the patients treated with SOF/LDV and those with ASV/DCV. If it lacks the numerical data for mean and standard deviation, we use median, range, and sample size to estimate the mean and variance [19]. If the median and interquartile range (IQR) is reported, we assume that the median is equal to the mean and width of the interquartile range is approximately 1.35 times of standard deviation [20]. Difference in means with 95% CI were calculated for each study and for those studies combined. A χ^2^-based test of homogeneity was performed and the inconsistency index (I^2^) and Q statistics were determined. If I^2^ statistic were > 50%, a random-effects model (DerSimonian-Laird method) was used. Otherwise, a fixed-effects model (Mantel–Haenszel method) was employed. Combined effects were calculated and a 2-sided P value < 0.05 was considered to indicate statistical significance. Sensitivity analyses were performed using the leave-one-out method in which the meta-analysis was performed with each study removed in turn. Publication bias was assessed by constructing a funnel plot by Egger’s test. The absence of publication bias was indicated by the data points forming a symmetric funnel-shaped distribution and one-tailed significance level of P > 0.05 (Egger’s test). All analyses were performed using Comprehensive Meta-Analysis statistical software, version 2.0 (Biostat, Englewood, NJ, USA).

## Results

### Literature search

**Fig. 1:**
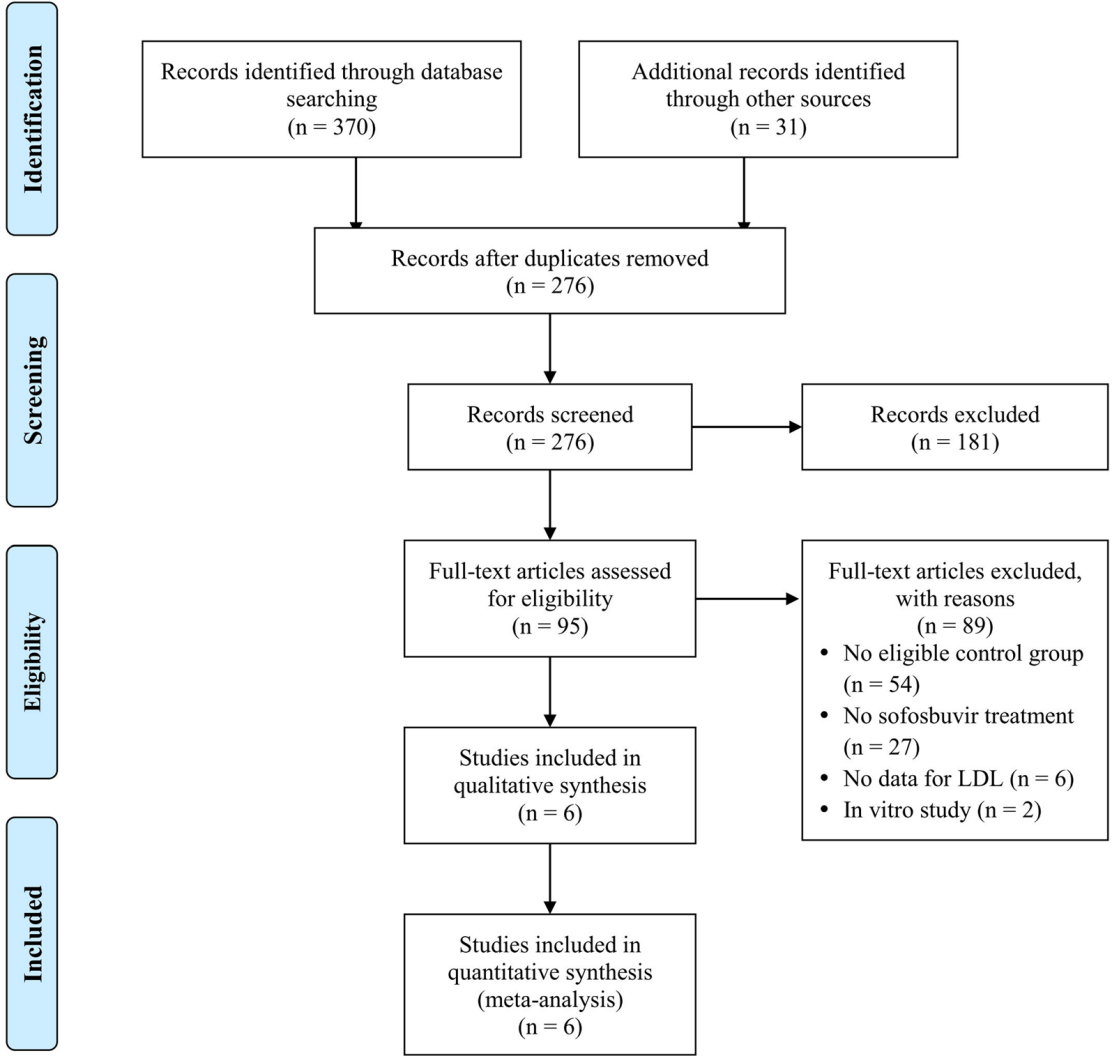
Flowchart of study selection

The process of study selection was presented in Fig. 1. After initially identifying 276 records, 181 records were excluded and 95 articles were left for full-text review. Eighty-nine articles were excluded after reviewing the full-text articles. The reasons for exclusion were provided in Fig. 1. The remaining six studies were included for subsequent qualitative and quantitative analyses [21–26].

### Study characteristics

A total of 1248 patients with HCV infection were encompassed by the six studies: 848 patients were treated with SOF-based DAAs and 400 patients with non-SOF DAAs (Tables 1, 2). Mean age of patients ranged from 62 to 76 years and the percentage of male ranged from 32.3 to 85.7%. Only two study included the patient with genotype 2 [23, 26]. Two studies only analyzed the patients achieving SVR12 [21, 24], three studies achieved good results of SVR [23, 25, 26], and Hashimoto et al. did not report the outcome of SVR [22].

**Table 1:** Baseline characteristics of the included studies

First author (publication year)	Study design	No of patients	Group	Age (year)	Male (%)	Genotype (%)	SVR (%)	Fibrosis-4 index	HCV-RNA (log IU/mL)	Baseline LDL (mg/dL)	Exclusion criteria
Özdoğan (2020)	NA	93	SOF/LDV	64.1 (12.8)^a^	32.3	1: 96.7 2: 2.5 4: 0.8	SVR12: 98.4	NA	3.5 (10.0)^a^	81.7 (28.4)^a^	HIV or HBV infection, HCC, liver transplant, patients taking lipid-lowering agents
		28	PTVr/OBV + DSV	64.1 (14.3)^a^	85.7				3.0 (7.5)^a^	89.7 (26.2)^a^	
Inoue (2018)	NA	85	SOF/LDV	64.4 (13.3)^a^	43.5	1b: 100	SVR24: 97.8	3.4 (2.4)^a^	NA	102.8 (25.5)^a^	NA
		46	SOF/RBV	62.0 (15.3)^a^	32.6	2: 100	SVR24: 98.8	3.5 (2.8)^a^		103.2 (28.0)^a^	
		85	ASV/DCV	68.3 (10.5)^a^	38.8	1b: 100	SVR24: 81.2	4.3 (2.9)^a^		98.3 (29.0)^a^	
Endo (2017)	Retrospective cohort study	132	SOF/LDV	66.7 (13.1)^a^	37.1	1b: 100	SVR12: 100	4.7 (4.0)^a^	5.9 (0.87)^a^	89.2 (28.5)^a^	HIV infection, HCC, decompensated cirrhosis
		121	ASV/DCV	68.4 (11.8)^a^	48.8	1b: 100	SVR12: 100	4.9 (3.3)^a^	5.9 (0.78)^a^	82.8 (27.2)^a^	
Kan (2017)	NA	55	SOF/LDV	70.0 (20–86)^c^	56.4	1a/1b: 9/91	SVR12: 100	47.3% (≥ 3.25)	6.1 (2.5–7.2)^c^	77.0 (34–139)^c^	Decompensated cirrhosis, patients taking lipid-lowering agents
		40	PTVr/OBV	67.0 (46–85)^c^	72.5	1b: 100	SVR12: 100	55.0% (≥ 3.25)	6.2 (3.9–7.3)^c^	75.5 (38–134)^c^	
Notsumata (2017)	NA	140	SOF/LDV	71.0 (26–87)^c^	40.2	NA	SVR12: 99.1	NA	NA	90.2 (22.9)^a^	NA
		100	SOF/RBV				SVR12: 98.7			98.1 (25.3)^a^	
		173	ASV/DCV				SVR12: 93.8			88.7 (30.0)^a^	
		50	PTVr/OBV				SVR12: 96.3			101.3 (24.4)^a^	
Hashimoto (2016)	Retrospective cohort study	76	SOF/LDV	69.0 (63.0- 75.0)^b^	40.8	1: 100	NA	67.1% (≥ 3.25)	6.0 (5.6–6.4)^b^	87.5 (68.6–105.8) ^b^	Patients taking lipid-lowering agents
		24	ASV/DCV	76.0 (69.0- 80.0)^b^	33.3	1: 100	NA	79.2% (≥ 3.25)	6.1 (5.4–6.4)^b^	80.2 (65.3–94.6)^b^	

Data were presented as mean (standard deviation)^a^, median (interquartile range)^b^, or median (range)^c^

*LDL* low-density lipoprotein; *SVR* sustained virologic response; *SOF* sofosbuvir; *LDV* ledipasvir; *DCV* daclatasvir; *ASV* asunaprevir; *PTVr* paritaprevir/ritonavir; *OBV* ombitasvir; *RBV* ribavirin; *HIV* human immunodeficiency virus; *HCC* hepatocellular carcinoma; *NA* not available

**Table 2:** Summary of regimens of the included studies

First author (publication year)	No of patients	Group	Regimens	Duration of treatment (week)
Özdoğan (2020)	93	SOF/LDV	NA	12
	28	PTVr/OBV + DSV		12
Inoue (2018)	85	SOF/LDV	Harvoni® (SOF 400 mg + LDV 90 mg) QD	12
	46	SOF/RBV	Sovaldi® (SOF) 400 mg QD + Rebetol® (RBV) 600/800/1000 mg (depending on body weight) BID	12
	85	ASV/DCV	Dacluinza® (DCV) 60 mg QD + Sunbepra® (ASV) 100 mg BID	24
Endo (2017)	132	SOF/LDV	Harvoni® (SOF 400 mg + LDV 90 mg) QD	12
	121	ASV/DCV	Dacluinza® (DCV) 60 mg QD + Sunbepra® (ASV) 100 mg BID	24
Kan (2017)	55	SOF/LDV	Harvoni® (SOF 400 mg + LDV 90 mg) QD	12
	40	PTVr/OBV	Viekirax® (PTVr 150/100 mg + OBV 25 mg) QD	12
Notsumata (2017)	140	SOF/LDV	NA	12
	100	SOF/RBV		12
	173	ASV/DCV		24
	50	PTVr/OBV		12
Hashimoto (2016)	76	SOF/LDV	Harvoni® (SOF 400 mg + LDV 90 mg) QD	12
	24	ASV/DCV	ASV 100 mg + DCV 60 mg BID	24

*NS* nonstructural proteins; *NI* non-nucleoside inhibitors; *PI* protease inhibitors; *SOF* sofosbuvir; *LDV* ledipasvir; *ASV* asunaprevir; *DCV* daclatasvir; *PTVr* paritaprevir/ritonavir; *OBV* ombitasvir; *RBV* ribavirin; *QD* once daily; *BID* twice daily; *NA* not available

### SOF-based DAAs vs. non-SOF DAAs

**Fig. 2:**
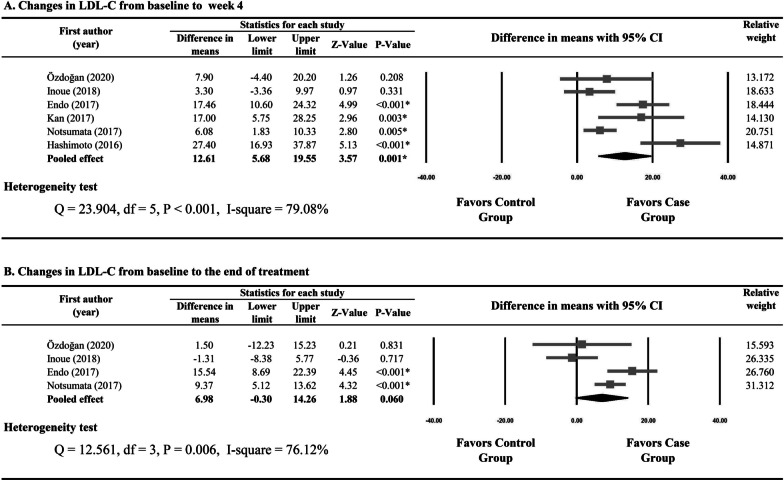

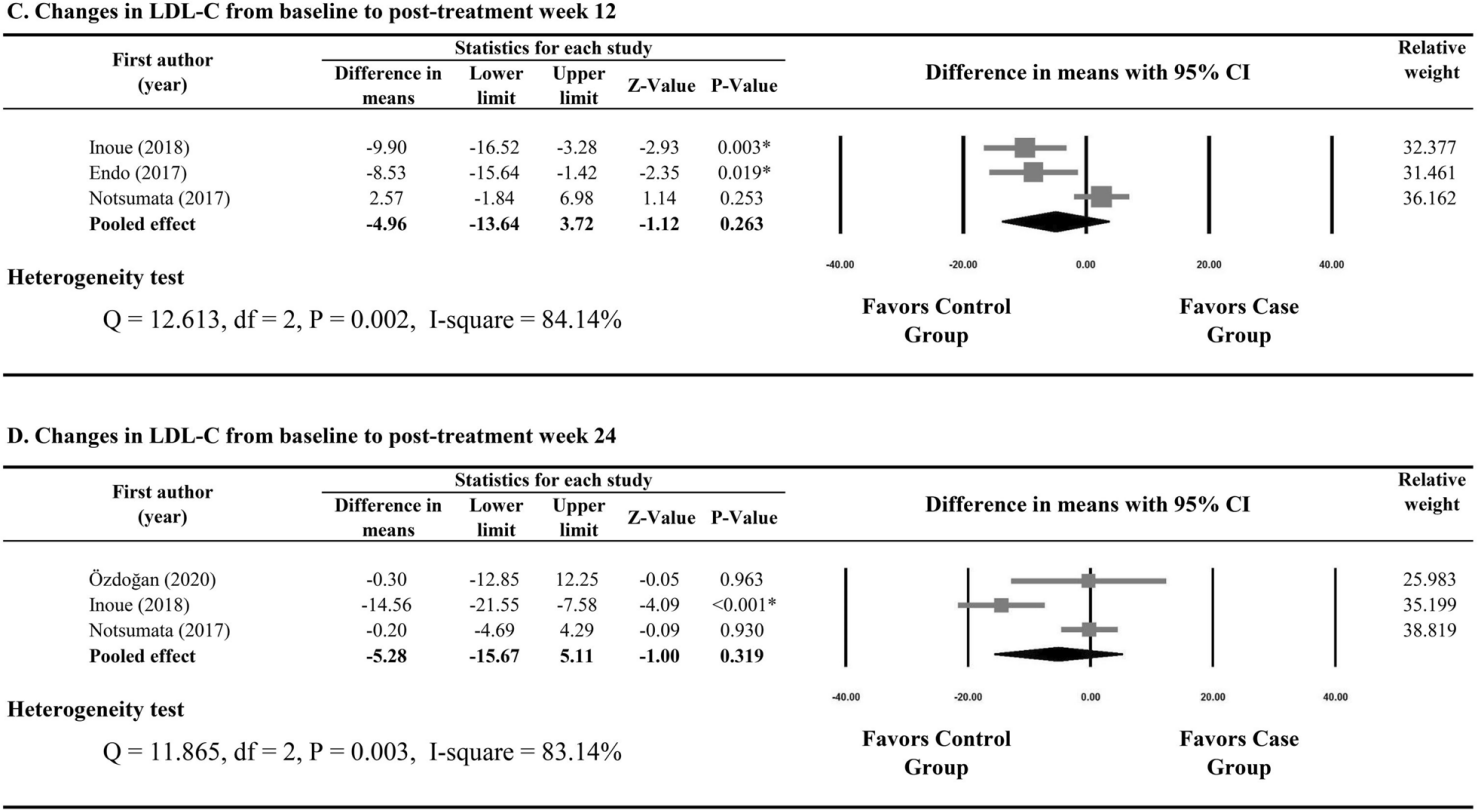
Forest plots for changes in low-density lipoprotein from baseline to week 4 (**A**), to the end of treatment (**B**), to post-treatment week 12 (**C**), and to post-treatment week 24 (**D**) between patients treated with sofosbuvir-based and those with non-sofosbuvir direct-acting antivirals

All the six included studies reported numerical data for changes in LDL from baseline to week 4 [21–26]. There was evidence of heterogeneity among the six studies (Q statistic = 23.904, I^2^ = 79.08%, P < 0.001); therefore, a random-effects model of analysis was used. Combined difference in means (12.61, 95% CI 5.68 to 19.55) indicated that the increases in LDL from baseline to week 4 were significantly greater in the SOF-based DAAs group than in the non-SOF DAAs group (P = 0.001) (Fig. 2A).

Four studies provided data for changes in LDL from baseline to the end of treatment [21, 23, 25, 26]. Because of evident heterogeneity (Q statistic = 12.561, I^2^ = 76.12%, P = 0.006), a random-effects model of analysis was used. The results indicated that the SOF-based DAAs group had greater increases in LDL from baseline to the end of treatment than the non-SOF DAAs group (6.98, 95% CI − 0.30 to 14.26); however, no statistical significance was reached (P = 0.060) (Fig. 2B).

There was significant heterogeneity in the studies reporting changes in LDL from baseline to post-treatment week 12 and from baseline to post-treatment week 24 (I^2^ = 84.14% and 83.14%, respectively). Meta-analysis showed that the SOF-based DAAs and non-SOF DAAs groups had similar changes in LDL from baseline to post-treatment week 12 (P = 0.263) and from baseline to post-treatment week 24 (P = 0.319) (Fig. 2C, D).

### SOF/LDV vs. ASV/DCV

**Fig. 3:**
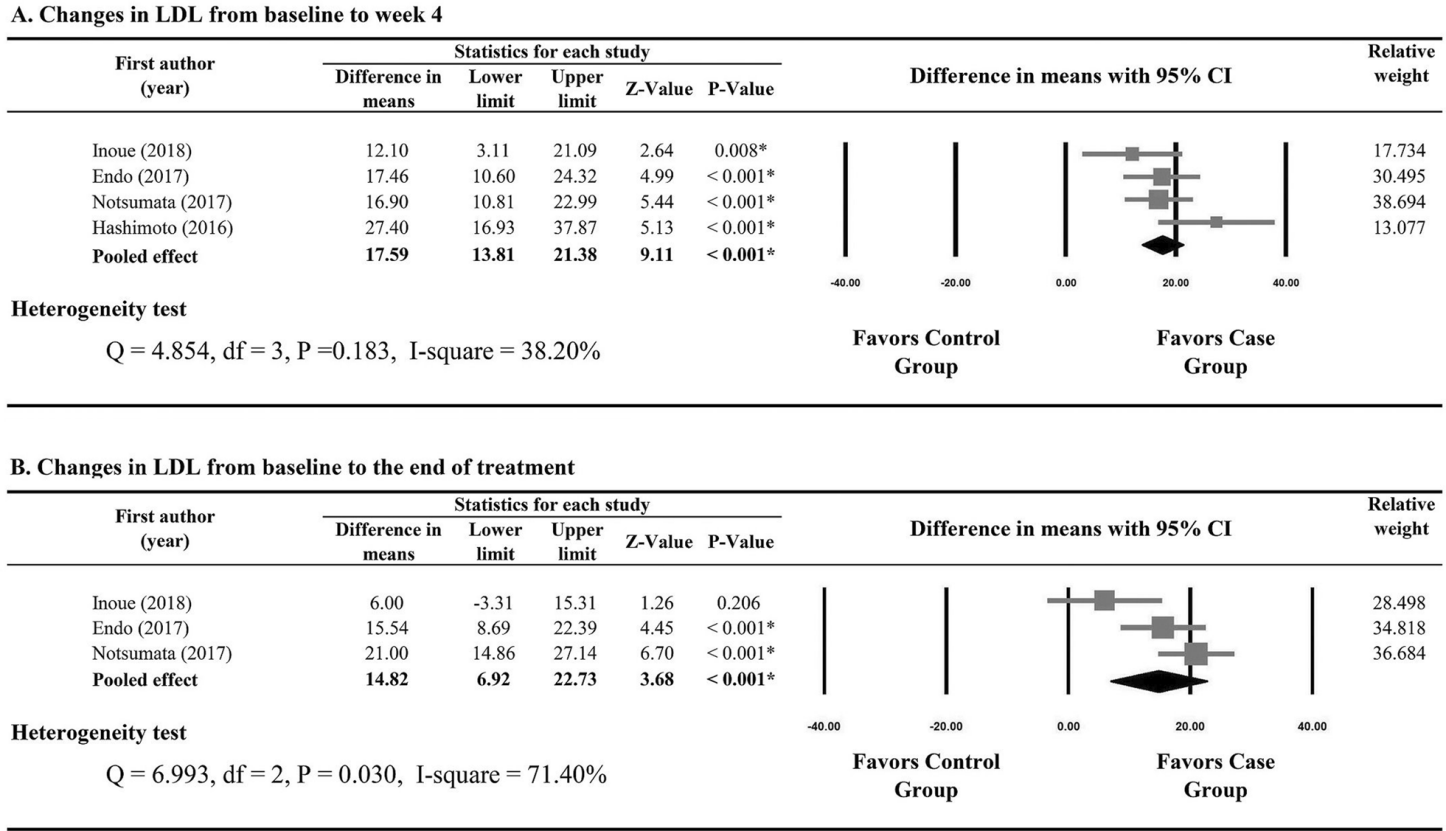

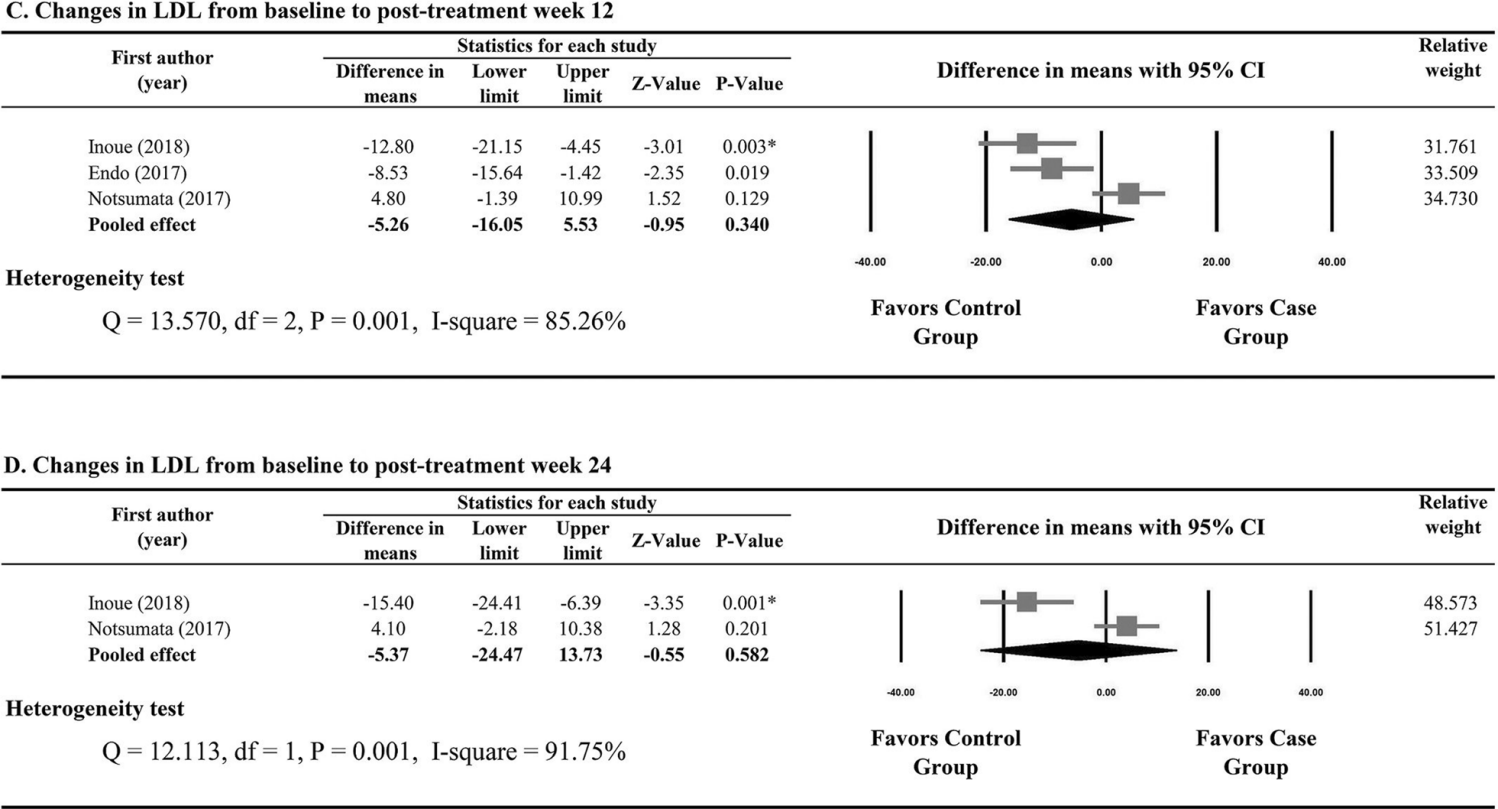
Forest plots for changes in low-density lipoprotein from baseline to week 4 (**A**), to the end of treatment (**B**), to post-treatment week 12 (**C**), and to post-treatment week 24 (**D**) between patients treated with sofosbuvir/ledipasvir and those with asunaprevir/daclatasvir

Four studies reported numerical data for changes in LDL from baseline to week 4 for the patients treated with SOF/LDV and those with ASVDCV [21–23, 25]. Due to non-significant heterogeneity of the four studies (Q statistic = 4.854, I2 = 38.20%, P = 0.183), a fixed-effects model was used and meta-analysis showed that the SOF/LDV group had greater increases in LDL from baseline to week 4 than the ASV/DCV group (17.59, 95% CI 6.92 to 22.73, P < 0.001) (Fig. 3A).

Numerical data for changes in LDL from baseline to the end of treatment between the patients treated with SOF/LDV and those with ASV/DCV were provided by three studies [21, 23, 25]. A random-effects model was used for the evident heterogeneity of the three studies (Q statistic = 13.570, I^2^ = 85.26%, P = 0.001). Pooled difference in means (14.82, 95% CI 6.92 to 22.73, P < 0.001) indicated that the increases in LDL from baseline to the end of treatment were significantly greater in the SOF/LDV group than in the ASV/DCV group (Fig. 3B).

Significant heterogeneity was observed in the studies reporting changes in LDL from baseline to post-treatment week 12 and from baseline to post-treatment week 24 (I^2^ = 85.26% and 91.75%, respectively). Results of meta-analysis showed that the SOF/LDV group and the ASV/DCV group had similar changes in LDL from baseline to post-treatment week 12 (P = 0.340) and from baseline to post-treatment week 24 (P = 0.582) (Fig. 3C, D).

### Sensitivity analysis

Table 3 presented the results of sensitivity analyses. For changes in LDL from baseline to week 4 between SOF-based DAAs and non-SOF DAAs groups, the direction and magnitude of combined estimates did not vary markedly with the removal of the studies, indicating that the meta-analysis had good reliability and the result was not overly influenced by each study. However, when we removed Inoue et al. the pooled difference in means for changes in LDL from baseline to the end of treatment became significant (P = 0.001). Moreover, when removing Notsumata et al. the pooled difference in means for changes in LDL from baseline to post-treatment week 12 became significant (P < 0.001). The results indicated that the pooled estimates for changes in LDL from baseline to the end of treatment might be affected by Inoue et al. and pooled estimates for changes in LDL from baseline to post-treatment week 12 might be affected by Notsumata et al. (Table 3). Regarding changes in LDL from baseline to post-treatment week 24, results of sensitivity analysis showed consistent direction and magnitude of combined estimates after removing any of the threes studies.

**Table 3:** Sensitivity analysis using leave-one-out method for changes in low-density lipoprotein

First author (publication year)	Statistics with study removed				
	Difference in means	Lower limit	Upper limit	Z-value	P-value
Sofosbuvir-based vs. non-sofosbuvir direct anti-viral agents					
Week 4					
Özdoğan (2020)	13.43	5.55	21.30	3.34	0.001
Inoue (2018)	14.79	6.75	22.84	3.60	< 0.001
Endo (2017)	11.54	3.76	19.33	2.91	0.004
Kan (2017)	11.93	4.20	19.66	3.02	0.002
Notsumata (2017)	14.39	5.76	23.02	3.27	0.001
Hashimoto (2016)	9.82	3.96	15.68	3.28	0.001
End of treatment					
Özdoğan (2020)	7.98	− 0.35	16.32	1.88	0.060
Inoue (2018)	10.41	4.51	16.32	3.45	0.001
Endo (2017)	3.93	− 4.16	12.01	0.95	0.341
Notsumata (2017)	5.62	− 6.54	17.77	0.91	0.365
Post-treatment week 12					
Inoue (2018)	− 2.61	− 13.47	8.24	− 0.47	0.637
Endo (2017)	− 3.41	− 15.62	8.80	− 0.55	0.584
Notsumata (2017)	− 9.27	− 14.11	− 4.42	− 3.75	< 0.001
Post-treatment week 24					
Özdoğan (2020)	− 7.12	− 21.19	6.94	− 0.99	0.321
Inoue (2018)	− 0.21	− 4.44	4.02	− 0.10	0.921
Notsumata (2017)	− 8.42	− 22.27	5.42	− 1.19	0.233
Sofosbuvir/ledipasvir vs. asunaprevir/daclatasvir					
Week 4					
Inoue (2018)	18.78	14.60	22.95	8.81	< 0.001
Endo (2017)	17.65	13.11	22.19	7.62	< 0.001
Notsumata (2017)	18.03	13.19	22.87	7.31	< 0.001
Hashimoto (2016)	16.12	12.06	20.18	7.78	< 0.001
End of treatment					
Inoue (2018)	18.49	13.15	23.82	6.79	< 0.001
Endo (2017)	13.92	− 0.75	28.60	1.86	0.063
Notsumata (2017)	11.31	2.02	20.60	2.39	0.017
Post-treatment week 12					
Inoue (2018)	− 1.75	− 14.81	11.32	− 0.26	0.793
Endo (2017)	− 3.77	− 21.01	13.47	− 0.43	0.668
Notsumata (2017)	− 10.33	− 15.74	− 4.91	− 3.74	< 0.001

Results of sensitivity analysis for changes in LDL between SOF/LDV group and ASV/DCV group were similar to those between SOF-based DAAs and non-SOF DAAs groups. The results of changes in LDL from baseline to week 4 between SOF/LDV group and ASV/DCV group were reliable. However, the results of changes in LDL from baseline to the end of treatment and from baseline to post-treatment week 12 were significantly influenced by the Inoue et al. and by Notsumata et al. respectively.

### Risk of bias

The results of risk of bias assessment were summarized in Table 4. According to ROBINS-I, two studies were judged to be at serious overall risk of bias [23, 25], two studies at moderate overall risk of bias [21, 26], and two studies at low overall risk of bias [22, 24]. The most significant risk of bias came from the domain of bias due to confounding. In the study by Inoue et al. the baseline lipid profiles were significantly different between treatment groups [23]. Notsumata et al. did not described the baseline characteristics regarding lipid profiles and medical histories [25]. Although Endo et al. included the patients who were taking lipid-lowering drugs and anti-diabetic drugs and did not control for these variables, serious residual confounding was not likely because these variables were balanced among the treatment groups [21]. Three studies excluded the patients who were taking lipid-lowering drugs and baseline lipid profiles were similar between groups [22, 24, 26]. Notably, only one study described the previous treatment history (i.e., treatment-naïve or treatment-experienced) [26] and only two studies clearly stated their study design [21, 22]. Two studies did not report detail regiments for each treatment group and was judged as moderate risk of misclassification bias [25, 26], whereas the other four studies as low risk of misclassification bias (Table 2). All the six studies were judged to be low risk in the domains of selection bias, performance bias, measurement bias, and reporting bias. All the six studies did not report any information about missing data.

**Table 4:** Risk of bias of the included studies

First author (publication year)	Bias due to confounding	Bias in selection of participants into the study	Bias in classification of interventions	Bias due to deviations from intended interventions	Bias due to missing data	Bias in measurement of outcomes	Bias in selection of the reported result	Overall risk of bias
Özdoğan (2020)	Low	Low	Moderate	Low	No information	Low	Low	Moderate
Inoue (2018)	Serious	Low	Low	Low	No information	Low	Low	Serious
Endo (2017)	Moderate	Low	Low	Low	No information	Low	Low	Moderate
Kan (2017)	Low	Low	Low	Low	No information	Low	Low	Low
Notsumata (2017)	Serious	Low	Moderate	Low	No information	Low	Low	Serious
Hashimoto (2016)	Low	Low	Low	Low	No information	Low	Low	Low

### Publication bias

The funnel plot showed that there was no publication bias for the outcome of the changes in LDL from baseline to week 4 via Egger’s test (t = 1.451, P = 0.110, Additional file 1: Fig. S1). For the other outcomes, the changes in LDL from baseline to the end of treatment, post-treatment week 12, and post-treatment week 24, publication bias analyses were not performed because the number of studies was too few to detect an asymmetric funnel [27].

## Discussion

To the best of our knowledge, this study is the first systematic review and meta-analysis to evaluate the effect of SOF-based DAAs on changes in lipid profiles. Our results showed that, compared with non-SOF DAAs, SOF-based DAAs was associated with rapid increases in LDL during the initial 4 weeks of treatment and the changes did not sustain after the end of treatment. A further comparison of SOF/LDV with ASV/DCV also revealed a similar trend. Therefore, close monitoring patients with rapidly rising LDL levels during and after DAAs treatment, instead of the use of lipid-lowering agents, could be considered.

The potential mechanism underlying the changes in LDL with DAAs has not been clearly elucidated. Because the included patients in this meta-analysis were mainly those who achieved SVR, viral clearance could not account for the different changes in LDL between SOF-based DAAs and non-SOF DAAs. As a nucleotide prodrug, SOF is converted to active compounds by enzymatic cleavage of phosphoramidate side chain, and then is decomposed to GS-060965, phenolate ion, and propan-2-yl 2-aminopropanoate in hepatocytes (Additional file 2: Fig. S2) [28]. Another drug with a similar structure and metabolic pathway to SOF is tenofovir alafenamide (TAF), a prodrug of tenofovir for the treatment of HIV and chronic hepatitis B. Several studies also reported an increased level of LDL with TAF. Both the study by Milinkovic et al. and by Taramasso et al. observed that LDL levels significantly increased in HIV patients after switching from tenofovir disoproxil fumarate (TDF) to TAF [29, 30]. In the study by Cid-Silva et al. TAF was associated with a more significantly increased level of LDL than TDF [31]. Notably, both SOF and TAF have a similar structure of phosphoramidate side chain and have impacts on lipid profiles, whereas TDF does not (Additional file 3: Fig. S3). We presumed that the cleaved products from phosphoramidate side chain might be the key agents, which would promote β-lipoprotein synthesis and secretion in hepatocytes and then be enzymatically metabolized. Consequently, a rapidly rising and gradually falling level of LDL was observed. However, further investigations are warranted for proving the hypothesis.

Although our results were comparable with some reports that the increases in LDL disappeared after treatment [32, 33], other studies suggested the elevated LDL continued to post-treatment 1 year [34–37]. The main reason for the inconsistency was that most of these studies were single-arm studies. Without a control group, it is difficult to judge the effect of SOF-based DAAs on changes in LDL. Additionally, both Younossi et al. and Pedersen et al. observed that genotype 3 patients had significantly increased LDL during DAAs treatment, but genotype 1 or genotype 2 patients did not [38, 39]. As the majority of the included patients in the present study were genotype 1 and genotype 2 patients (Table 1), changes in LDL would be less, consequently reducing the difference between treatment groups. Furthermore, genetic factors have been reported in association with changes in LDL. In the study by Emmanuel et al. the *IFNL4-ΔG* carriers had significant increases in LDL during DAAs treatment and at post-treatment 1 year, but the patients with *IFNL4-TT/TT* did not [40]. In the study by Morihana et al. the difference in LDL between SOF/LDV and ASV/DCV disappeared after the end of treatment. However, the *IL28B TG/GG* patients continued to have increased LDL from the end of treatment to post-treatment two years, whereas the *IL28B TT* patients did not [32]. Because this meta-analysis did not consider these genetic factors, our results might be potentially confounded by these predictors.

There were several limitations in this study. The first limitation came from baseline confounding bias as our results demonstrated evident heterogeneity among the included studies. Secondly, most of the included patients achieved SVR. Although it has been noted that SVR patients had significantly greater increases in LDL with DAAs than non-SVR patients, it should be cautious in interpreting our results [15, 41]. Thirdly, due to a lack of sufficient numerical data, we only compared SOF/LDV with ASV/DCV among the numerous DAAs regimens, did not assess the changes in total cholesterol, triglycerides, high-density lipoprotein, and apolipoprotein, and did not evaluate the longer-term changes in LDL. Only one of the included reported the outcomes at post-treatment 1 year [26]. It deserves more comparative studies, which evaluate complete lipid profiles at different time points among DAAs regimens. Finally, due to the limited number of the included studies (n = 6), we only performed subgroup analysis of SOF/LDV vs. ASV/DCV and no additional analysis could be done, for example, subgroup analyses based on study characteristics or meta-regression analysis. Due to the same reason, analysis of publication bias was not performed for all outcomes. As Sutton et al. recommended the minimum requirement of ten studies for publication bias analysis, it should be cautious in interpreting our results [27].

## Conclusions

For HCV patients, SOF-based DAAs rapidly and significantly increased LDL level during the initial 4 weeks of treatment, but changes in LDL tended to disappear at and after the end of treatment. Potential mechanism underlying changes in lipid profiles with DAAs treatment might be related with the cleaved products of phosphoramidate side chain of SOF and deserves more investigations.

## Supplementary Information

**Additional file 1: Fig. S1.** Funnel plot forchanges in low-density lipoprotein from baseline to week 4. 

**Additional file 2: Fig. S2.** Decompositionpathway of sofosbuvir. In hepatocytes, the phosphoramidate side chain of sofosbuvir is enzymatically cleaved into phenolateion, propan-2-yl 2-aminopropanoate, and nucleotide GS-606965 which is furtherphosphorylated to an active metabolite GS-461203.  

**Additional file 3: Fig. S3.** Chemicalstructures of tenofovir alafenamide, sofosbuvir, and tenofovir disoproxilfumarate. Tenofovir alafenamide and sofosbuvir have a similar structure of phosphoramidate sidechain.

## Data Availability

All data relevant to the study are included in the article.

## Abbreviations

ASV: Asunaprevir
DAAs: Direct-acting antiviral
DCV: Daclatasvir
HCV: Hepatitis C virus
LDL: Low-density lipoprotein
LDV: Ledipasvir
NS: Nonstructural protein
RNA: Ribonucleic acid
ROBINS-I: Risk Of Bias In Non-randomized Studies- of Interventions
SVR: Sustained virologic response
TAF: Tenofovir alafenamide
TDF: Tenofovir disoproxil fumarate
VLDL: Very low-density lipoprotein
