# Supplementary figures and images for: Effect of sofosbuvir-based DAAs on changes in lower-density lipoprotein in HCV patients: a systematic review and meta-analysis
Source: BMC Infect Dis. 2021 Sep 21;21:984. doi: 10.1186/s12879-021-06657-9 (PMC8454153; doi:10.1186/s12879-021-06657-9)

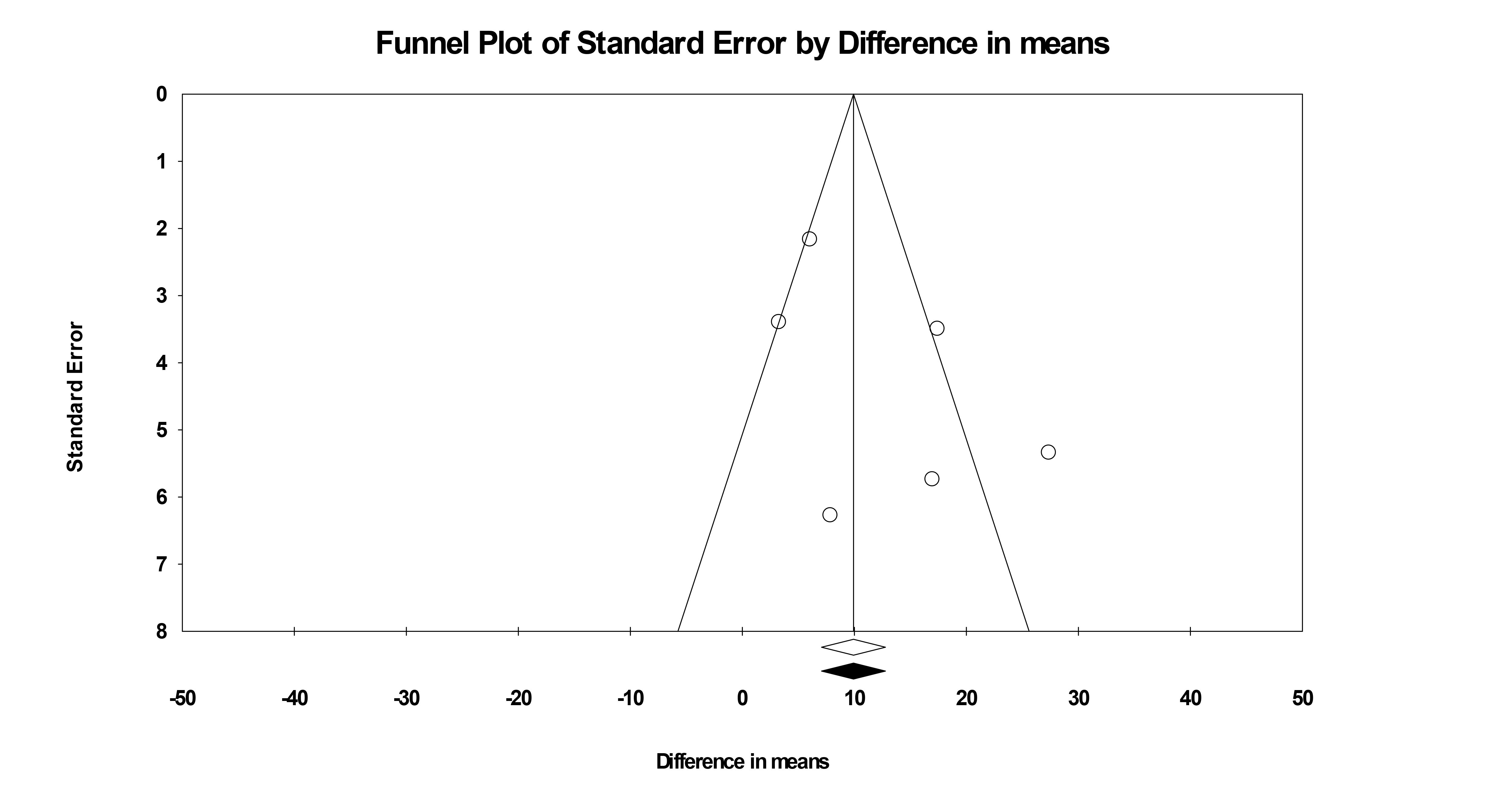

Supplement: Supplementary file 1 — Additional file 1: Fig. S1. Funnel plot forchanges in low-density lipoprotein from baseline to week 4. [file 12879_2021_6657_MOESM1_ESM.jpg]

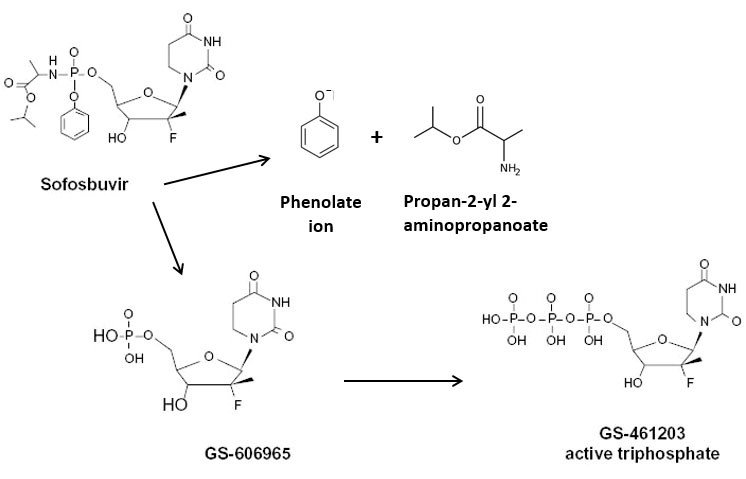

Supplement: Supplementary file 2 — Additional file 2: Fig. S2. Decompositionpathway of sofosbuvir. In hepatocytes, the phosphoramidate side chain of sofosbuvir is enzymatically cleaved into phenolateion, propan-2-yl 2-aminopropanoate, and nucleotide GS-606965 which is furtherphosphorylated to an active metabolite GS-461203. [file 12879_2021_6657_MOESM2_ESM.jpg]

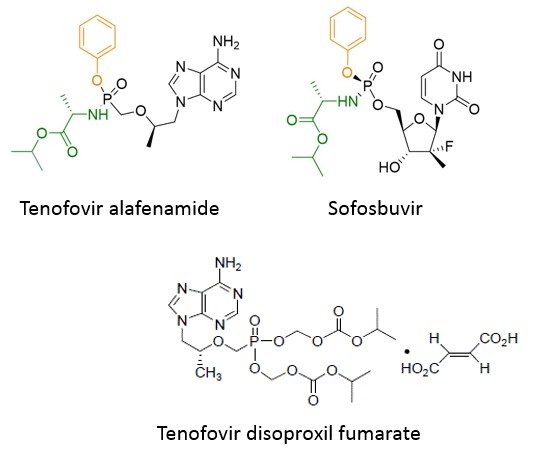

Supplement: Supplementary file 3 — Additional file 3: Fig. S3. Chemicalstructures of tenofovir alafenamide, sofosbuvir, and tenofovir disoproxilfumarate. Tenofovir alafenamide and sofosbuvir have a similar structure of phosphoramidate sidechain. [file 12879_2021_6657_MOESM3_ESM.jpg]
